# Matrix Effects on the Microcystin-LR Fluorescent Immunoassay Based on Optical Biosensor

**DOI:** 10.3390/s90403000

**Published:** 2009-04-23

**Authors:** Feng Long, An-na Zhu, Jian-Wu Sheng, Miao He, Han-Chang Shi

**Affiliations:** 1 Environmental Simulation and Pollution Control State Key Joint Laboratory, Dept. of Environment Science and Engineering, Tsinghua University, Beijing 100084, P.R. China; E-Mails: longf04@mails.thu.edu.cn; shengjw@mail.tsinghua.edu.cn; hemiao@mail.tsinghua.edu.cn;; 2 Research Institute of Chemical Defence, Beijing 102204, P.R. China; E-Mail: zhuanna00@mails.thu.edu.cn

**Keywords:** Matrix effects, Microcystin-LR, fluorescent immunoassay, optical biosensor

## Abstract

Matrix effects on the microcystin-LR fluorescent immunoassay based on the evanescent wave all-fiber immunosensor (EWAI) and their elimination methods were studied. The results indicated that PBS and humic acid did not affect the monitoring of samples under the investigated conditions. When the pH was less than 6 or higher than 8, the fluorescence signals detected by immunosensor systems were obviously reduced with the decrease or increase of pH. When the pH ranged from 6 to 8, IC_50_ and the linear working range of MC-LR calculated from the detection curves were 1.01∼1.04 μg/L and 0.12∼10.5 μg/L, respectively, which was favourable for an MC-LR immunoassay. Low concentrations of Cu^2+^ rarely affected the detection performance of MC-LR. When the concentration of CuSO_4_ was higher than 5 mg/L, the fluorescence signal detected by EWAI clearly decreased, and when the concentration of CuSO_4_ was 10 mg/L, the fluorescence signal detected was reduced by 70%. The influence of Cu^2+^ on the immunoassay could effectively be compromised when chelating reagent EDTA was added to the pre-reaction mixture.

## 1. Introduction

Evaluation of matrix effects is of great importance when developing a quantitative immunoassay method because antigen and antibody binding depends mainly on van der Waals forces and hydrophobic interactions, which are greatly affected by effects existing in real water samples such as pH, ionic strength, organic content and so on [1]. Matrix effects may be defined as ‘the sum of the effects of all of the components, qualitative or quantitative, in a system with the exception of the analyte to be measured’ [1,2]. Environmental samples are usually comprised of extremely complex and variable mixtures of proteins, carbohydrates, lipids, small molecules, salts and so on, which may lead to false positive or false negative results, low or high bias or poor precision. The reagents used in the immunoassay may also cause matrix effects. Despite enormous advances in the design of immunoassays, unwanted interferences caused by matrix effects cannot be completely excluded. Moreover, the interferences in the measurement of different samples usually vary with each other, so when detecting a target pollutant of interest with an immunosensor, special methods for eliminating matrix effects must often be used to obtain correct assay results.

Microcystin-LR (MC-LR) containing l-leucine and r-arginine in positions 2 and 4, respectively, is the most frequent and most toxic among nearly 80 microcystin variants obtained from *Microcystis, Anabaena, Oscillatoria (Planktothrix), Nostoc* and *Anabaenopsis* [3]. Many reported cases of animal-poisoning and human health diseases, some resulting in liver cancer and even death, are due to exposure to MCs via drinking and surface water [4–6]. To minimize public exposure to MCs, the World Health Organization (WHO) has proposed a drinking water MC-LR guideline value (GV) of 1 μg/L [3]. Some immunoassay technologies have been developed to detect MC-LR [7,8], but due to the matrix interferences in water samples, most of them could not be applied to assay the real samples [9]. Fluorescent immunosensors have been developed to determine various trace amounts of targets interest based on the principle of fluorescent immunoassay [10–12]. However, a detailed evaluation of common organic and inorganic substances found in the environment for the detection of MC-LR based on fluorescent immunosensor is still missing. We have previously introduced a new portable miniaturized evanescent wave all-fiber immunosensor (EWAI) to determine various trace amounts of targets interest based on the principle of immunoreaction and total internal reflect fluorescent (TIRF) [13]. Here we use the slightly revised EWAI to investigate the influence of common interferences like PBS, pH, humic acid and copper ions on the sensitivity and stability of the MC-LR fluorescence immunoassay, and demonstrated that with the choice of a proper elimination method, the influence of interfering substances can be limited.

## 2. Experimental

### 2.1. Immunoreagents and Chemicals

3-mercaptopropyl-trimethoxysilane (MTS), ovalbumin (OVA), bovine serum albumin (BSA), *N*-(4-maleimidobutyryloxy) succinimide (GMBS), and 1-ethyl-3-(dimethylaminopropyl) carbodiimide hydrochloride (EDC) were purchased from Sigma-Aldrich (Steinheim, Germany). MC-LR was obtained from Alexis (Lausen, Switzerland). All the other reagents, unless specified, were supplied by Beijing Chemical Agents; these were also of analar grade and used without further purification. Distilled deionized water was used throughout the investigation. Monoclonal anti-MC-LR antibody (MC-LR-MAb. reference no. 8C10) was produced and the hapten conjugate MC-LR-OVA was synthesized by our research group. 1×PBS was 0.01 mol/L phosphate buffer, 0.8% saline solution and unless otherwise indicated the pH was 7.4. 5×PBS and 10×PBS is 5 times and 10 times concentrated 1×PBS. 1 mg/L MC-LR stock solutions were prepared in 0.01 mol/L PBS and stored at 4 °C.

### 2.2. EWAI instrumentation

The slightly modified EWAI immunosensor used in this study was previously described in [13]. The pulse laser beam from a 635-nm pulse diode laser was directly launched into the single-mode fiber of the single-multi mode fiber coupler. The laser light then entered the multi-mode fiber with the diameter of 600 μm and numerical aperture of 0.22 from the single-mode fiber. Afterwards, the excitation light from the laser, through the fiber connector, was coupled to a fiber probe. The incident light propagates along the length of the probe via total internal reflection. The evanescent wave generated at the surface of the probe then interacted with the surface-bound fluorescently labelled analyte complexes, and causes excitation of the fluorophores. The collected fluorescence was subsequently filtered by means of a bandpass filter and detected by photodiodes through lock-in detection. The probe was embedded in a flow glass cell with a flow channel having a nominal dimension of 70 mm in length and 2 mm in diameter. All reagents were delivered by a flow analysis system operated with a peristaltic pump.

### 2.3. Probe preparation

Combination tapered fiber optic probes were prepared as previously described [14]. The hapten-carrier conjugate MC-LR-OVA, used as recognition element, were covalently attached to the sensing surface of the probes with a heterobifunctional reagent. Employing a modified procedure originally described by Bhatia *et al*. [15], the hapten-carrier conjugate was immobilized onto the probe surface. Briefly, the probes were initially cleaned with piranha reagents (concentrated H_2_SO_4_/H_2_O_2_ 2:1), rinsed with distilled deionized water, and dried in N_2_. Next, the probe was placed in 2% MTS in toluene for 2 hours, under an inert atmosphere. Excess MTS was eliminated with dry toluene to assure the order and uniformity of the SAM. The thiol group of the silane was allowed to react for 1 hour with a heterobifunctional crosslinker, 2 mM GMBS in ethanol. After rinsing with ethanol and PBS, the succinimide group on the GMBS was then used to covalently bind the epsilon amino groups on proteins. Immersion of the probe for 20 min in 2 mg/mL BSA was then carried out to block its non-specific binding sites.

### 2.4. Immunoassay procedure

The indirect competitive inhibition method was developed for MC-LR determination. Free analyte (MC-LR, 240 μL) of different concentrations was mixed with a fixed (0.6 μg/mL) concentration of antibody in PBS (240 μL) supplemented with BSA (2.0 mg/mL), which reduce non-specific binding of antibody, and allowed for incubation at room temperature for 6 min. Then, this mixture solution was delivered over the MC-LR-OVA immobilized fiber-optic probe surface at 240 μL/min in 2 min. After the other 6 min reaction, regeneration of the sensor surface was carried out with 2 mg/mL pepsin (pH 1.9) for 4 min followed by a short pulse (15 s) of acetonitrile, proprionic acid and water (50:1:50) and rinsing with PBS. The regeneration cycle was repeated twice in order to remove all antibodies remained on the surface. The whole immunoreactions’ process was on-time monitored by EWAI.

During pre-incubation, antibody binding sites were occupied depending on the concentration of the MC-LR. Only the antibodies left with free binding sites were able to bind to the antigen (MC-LR) immobilized onto the probe. Thus, as the amount of free MC-LR, the number of antibody available for interaction with MC-LR immobilized onto probe surface is decreased and vice versa. Based on this dependence, free MC-LR in the sample solution can be quantified. Real-time monitoring of the fluorescence signal was also undertaken as binding occurred between antibodies with free binding sites and the immobilized conjugate of the probe. All the assays were performed in triplicate.

### 2.5. Effect of the ionic strength

In immunoassay, PBS solution is usually used to prepare antibody or antigen standard solutions, and may affect the results of immunoassay. To evaluate the effect of PBS of different concentrations on the detection, 1xPBS, 5xPBS, 10xPBS were used to prepared the MC-LR standard solutions and Cy5.5-MC-LR-antibody solution, respectively.

### 2.6. Effect of the pH

We considered the effect of different pH on the MC-LR fluorescent immunoassay. 1×PBS solution was adjusted to different pH values with 1 mol/L HCl or NaOH, respectively. PBS solutions ranging from pH 3 to pH 11 were used to prepare solutions of the immunoreagents.

### 2.7. Effect of copper ion

The concentration of CuSO_4_ used for algal bloom removal generally ranges from 0.1 to 4 mg/L [16]. Therefore, the concentration of CuSO_4_ investigated in this work ranged from 0 to 10 mg/L. MC-LR samples were spiked with CuSO_4_ solution of different concentrations. After pre-reaction of the same amount of antibody and antigen for 10min, the mixture was delivered to the sample cell to detect. The measurements were performed at about pH 7.4. To eliminate the effect of high concentration CuSO_4_ on the immunoassay, 6 μL (1 mg/mL) EDTA was added to the mixture of antibody solution and MC-LR standard solutions, which were prepared using 5×PBS. And all the above experiments were repeated.

### 2.8. Effect of humic acid

The influence of the humic acid, which represent as humic substances, on the immunoassay performance was investigated. For this, MC-LR samples were spiked with humic acid sodium salt at concentrations ranging between 0 and 200 mg/L. Then, after pre-reaction of the same amount of antibody and antigen for 10 min, the mixture was delivered to the sample cell to detect.

## 3. Results and Discussion

### 3.1. Effect of the ionic strength

It has been reported that in a competitive immunoassay, the ionic strength may have a direct effect on the sensitivity of the immunoassay [17,18], but other studies stated that the ionic strength had no obviously effect on the immunoassay performance [19,20]. Therefore, we studied the effect of different concentrations of PBS on the sensitivity and stability of the EWAI.

**Figure 1.**
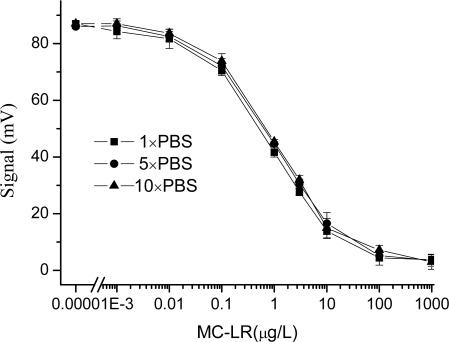
Effect of the ionic strength on the MC-LR immunoassay based on EWAI. Different concentrations of PBS (1×, 5×, and 10×) were used to prepare the standard solutions and antibody solutions and tested by using EWAI.

The experimental results are shown in Figure 1. The dynamic MC-LR detection range of the EWAI sensor is described to exhibit 20∼80% inhibition. From Figure 1, this meant that the linear working range of MC-LR was 0.12∼9.8 μg/L, 0.14∼10.2 μg/L, and 0.15∼10.5 μg/L (response to 1×PBS, 5×PBS, 10×PBS, respectively). The limit of detection (LOD) for MC-LR of 0.012, 0.019, and 0.024 μg/L (response to 1×PBS, 5×PBS, 10×PBS, respectively) were also calculated from the calibration curve as the analyte concentration providing a 10% decrease of the blank signal. On the other hand, no significant variations on the IC_50_ and Signal_max_ were observed by changing the ionic strength of the media. These suggested that the ionic strength did not affect the performance of EWAI under the indirect competitive detection mode. Due to buffer solutions may reduce the effect of other matrice (e.g. pH, and salt and so on) on the immunoassay, different concentrations of PBS may be used to prepare the samples and/or antibody solution in the assay of the real water samples.

### 3.2. Effect of the pH

**Figure 2.**
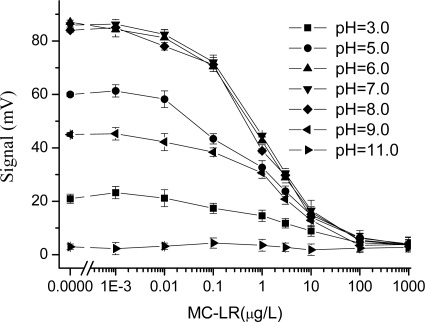
Effect of the pH on the MC-LR immunoassay based on EWAI. 1xPBS at different pH values were used to prepare the standard solutions and antibody solutions, respectively, and they were tested by using EWAI.

In an immunoassay, the pH of solutions obviously do not only affect the stability and biological activity of antibodies, but also the binding efficiency between antibody and antigen, which may lead to detection errors [21,22], if the effect of the pH is not taken into account. To evaluate this effect, detection curves of MC-LR were prepared in PBS buffer at pH values varying from 3.0 to 11.0. From Figure 2, at lower pH values or higher pH values, a decrease of the signal was observed in the EWAI determination of MC-LR. For example, when the pH was 3.0, the maximum EWAI signal was decreased by more than 70% than that in pH 7.0 in the absence of analyte. Moreover, the immunoassay was much more sensitive to basic than acidic conditions. Especially, above pH 11.0 no signal was obtained, indicating that fluorescent signal was not obtained by either non-specific adsorption of Cy5.5-MC-LR-MAb on the sensor surface, or excitation of free Cy5.5-MC-LR-MAb in the solution.

In contrast, between pH 6 and pH 8 the immunoassay was more sensitive, and almost no changes were observed on the immunoassay features including the linear working range, LOD, IC_50_, and Signal_max_. These results showed that pH value of analyte solution close to neutral environment (pH = 7.4) is the most favourable for the binding of antigen-antibody reaction in MC-LR immunoassay based on the EWAI. As a result, when the pH of the real water samples tested is too high or too low, a higher concentration of buffer solution (e.g. 5 × PBS or 10 × PBS) should be considered to prepare samples and antibody solution to eliminate its detriment to immunoassay.

### 3.3. Effect of copper ions and its elimination

Due to its biological activity toward algae, copper sulfate has been used since 1904 for nuisance algae control in surface waters [23]. Application of copper sulfate remains the most commonly used method for controlling nuisance algae in lakes and reservoirs [16], and therefore the eutrophicated lakes often contain potentially high levels of copper ions which may affect the results of MC-LR immunoassay.

**Figure 3.**
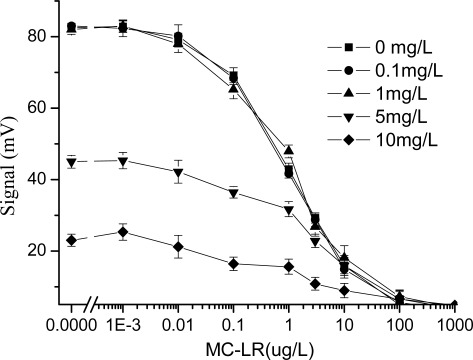
Effect of the copper ions on the MC-LR immunoassay based on EWAI (not containing EDTA in immunoassay system). The concentration of CuSO_4_ ranged from 0 to 10 mg/L.

In this study, the effect of copper ions, which were regarded as the representative of heavy metal ions, on the immunoassay is investigated. From Figure 3, the experimental results showed that when the concentration of CuSO_4_ was less than 1 mg/L, it rarely affected the detection results of MC-LR; and when that of CuSO_4_ was more than 1 mg/L, the concentration of copper ion was then increased and the decrease in signal was observed. When the concentration of CuSO_4_ is 5 mg/L, the signal response of the system was less than half of that in the absence of CuSO_4_. Especially, when the concentration of CuSO_4_ reached 10 mg/L, the system detected signals rapidly declined and could no longer be used. However, it was surprised why the response of EWAI was not the linear dependence on the copper concentration. Due to the effect of heavy metal ions on the immunoassay was complex process, we were difficult to explain this appearance under our experimental conditions.

Over the last years, there has been a growing interest in the immunnotoxicity of heavy ions. General consensus holds that the harmful effects of these ions mainly result from their interaction with proteins [24]. Studies showed that more than four Cu^2+^ ions per antibody molecule led to large insoluble aggregates [25], which may affect the performance of antibody. Although we could not make sure how many copper ions per antibody molecule led to large insoluble aggregates in this immunoassay system, when the concentration of copper ions was higher, part of them may chelate with or denature antibodies, and then led to the decrease of detection signals of EWAI.

**Figure 4.**
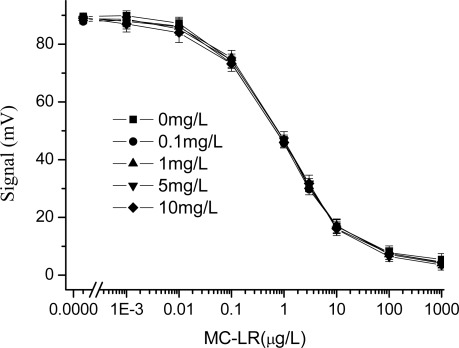
Effect of the copper ions on the MC-LR immunoassay based on EWAI (6 μL 1mg/mL EDTA added to the mixture). The concentration of CuSO_4_ ranged from 0 to 10 mg/L.

Therefore, in order to reduce the effect of the copper ion in samples on the detection results, 6 μL (1 mg/mL) of the chelating agent EDTA was added in MC-LR standard solutions containing different concentrations of CuSO_4_. And all the above experiments were repeated and the results, shown in Figure 4, stated that the effect of the copper ion obviously inhibited the immunoassay. The detection curves had better consistency, and the maximum signal value and IC_50_ did not differ significantly. The reason might be that copper ions in the solutions were chelated by EDTA and theirs harmful effect was compromised.

### 3.4. Effect of humic acid

It is well-known that the organic matter (dissolved and suspended) content in surface waters may have a negative effect on the MC-LR immune determination. The main mass of organic carbon distributed in natural aquatic environments is concentrated in humic substances. In general humic substances are the final products of microbial degradation processes of plants in waters. There is a lack of studies on the influence of naturally isolated humic substances on the performance of MC-LR immunoassay based on optical biosensor.

**Figure 5.**
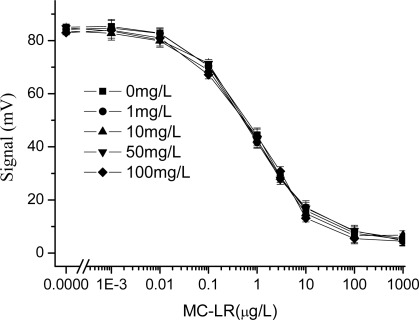
Effect of the humic acid on the MC-LR immunoassay based on EWAI. The concentration of humic acid ranged from 0 to 100 mg/L.

To evaluate this effect, detection curves of MC-LR were prepared in PBS containing different concentrations of humic acid, which is the representative of humic substances. As shown in Figure 5, the consistency of all standard curves is good, and the maximum signal value and IC_50_ did not differ significantly. Some studies have shown that there are a lot of different diameters hole in humic acid polymer to absorb or chemically react with organic pollutants and heavy metals [26]. Therefore, MC-LR molecule and antibody used in immunoassay may be absorbed by humic acid, which led to the decrease of the signal values detected. However, under the investigated conditions, the reason why the detected results were not affected might thank to that BSA added into the antibody solutions played a role in shielding in order to reduce non-specific adsorption of the antibody.

### 3.5. Water sample Analysis

Among the water samples tested were the laboratory tap water and two lake water samples collected from Lake Tai (Zhengjiang) and Lake Cao (Anhui), respectively. To eliminate the matrix effects, 240 μL different water samples were mixed with a 240 μL fixed (0.6 *μ*g/mL) concentration of antibody in 10×PBS supplemented with 1 mg/mL EDTA and 2.0 mg/mL of BSA. At first, the samples were measured unspiked. Then, the sample was added the standard MC-LR solution of different concentration and detected by EWAI. The results were shown in Table 1. Three individual experiments were tested for each sample.

From Table 1, the recovery of all measured samples was between 90 and 110%, and the parallel tests showed that the relativity coefficient was more than 0.992 (n=3). These results indicated that the possible interference from the different composition of water samples on EWAI analysis was negligible by adding PBS, EDTA and BSA in antibody solutions. The developed EWAI can be successfully applied to MC-LR analysis in real water samples.

## 4. Conclusions

In this research, some important areas of matrix interference in MC-LR immunoassay based on the EWAI and their elimination solutions have been discussed. It has not been possible to make detailed observations on every aspect due to the complexity and diversity of environmental matrices as well as the constraints of space. The experimental results of matrix effects indicated that PBS and humus under the scope of the experimental conditions did not affect the monitoring of samples, the proposed pH is 6∼8, and the influence of copper ions on the immunoassay could effectively be compromised when chelating reagent EDTA was added to the pre-reaction mixture.

## Figures and Tables

**Table 1.** Detection results of water sample.

**Origin**	**MC-LR in water sample (μg·L^−1^)**	**MC-LR added to the samples (μg·L^−1^)**	**MC-LR by EWAI (Mean) (μg·L^−1^)**	**CV (%)**	**Recovery (%)**
Tap water	0	0.5	0.45	2.1	90
		2	1.98	3.0	99
Tai Lake	0.52	0.5	0.96	1.2	96
		2	2.64	3.4	106
Cao Lake	0.31	0.5	0.83	1.5	104
		2	2.24	4.2	96.5
